# Physicochemical Properties, Antioxidant Capacity, and Bioavailability of *Laurus nobilis* L. Leaf Polyphenolic Extracts Microencapsulated by Spray Drying

**DOI:** 10.3390/foods12091923

**Published:** 2023-05-08

**Authors:** Erika Dobroslavić, Ivona Elez Garofulić, Zoran Zorić, Sandra Pedisić, Marin Roje, Verica Dragović-Uzelac

**Affiliations:** 1 Faculty of Food Technology and Biotechnology, University of Zagreb, Pierottijeva 6, 10000 Zagreb, Croatia; edobroslavic@pbf.hr (E.D.); ivona.elez@pbf.unizg.hr (I.E.G.); 2 Centre for Food Technology and Biotechnology, Faculty of Food Technology and Biotechnology, University of Zagreb, Petra Kasandrića 3, 23000 Zadar, Croatia; zzoric@pbf.hr (Z.Z.); spedisic@pbf.hr (S.P.); 3 Ruder Bošković Institute, Bijenička Cesta 54, 10000 Zagreb, Croatia; marin.roje@irb.hr

**Keywords:** laurel, polyphenols, microencapsulation, spray drying, bioaccessibility, antioxidant activity

## Abstract

Laurel (*Laurus nobilis* L.) leaves are a rich source of polyphenols with the potential for use in functional foods, where the main obstacle is their low stability and bioavailability, which can be improved by spray drying (SD). This research examined the influence of SD parameters, including inlet temperature (120, 150, and 180 °C), carrier type (β-cyclodextrin (β-CD); β-CD + maltodextrin (MD) 50:50; β-CD + gum arabic (GA) 50:50), and sample:carrier ratio (1:1, 1:2 and 1:3) on the physicochemical properties, encapsulation efficiency, polyphenolic profile, antioxidant capacity and bioaccessibility of laurel leaf polyphenols. The highest encapsulation efficiency was achieved at a sample:carrier ratio 1:2 and the temperature of 180 °C by using either of the applied carriers. However, the application of β-CD + MD 50:50 ensured optimal solubility (55.10%), hygroscopicity (15.32%), and antioxidant capacity (ORAC 157.92 μmol Trolox equivalents per g of powder), while optimal moisture content (3.22%) was determined only by temperature, demanding conditions above 150 °C. A total of 29 polyphenols (dominantly flavonols) were identified in the obtained powders. SD encapsulation increased the bioaccessibility of laurel flavonols in comparison to the non-encapsulated extract by ~50% in the gastric and ~10% in the intestinal phase, especially for those powders produced with carrier mixtures.

## 1. Introduction

Laurel (*Laurus nobilis* L.) is a Mediterranean shrub well known in folk medicine due to the many health-beneficial properties attributed mainly to the leaves extracts, which contain significant amounts of various polyphenols belonging to the groups of flavonols, flavan-3-ols, flavones, proanthocyanidins, and phenolic acids [1]. Laurel leaf polyphenols are known for their numerous biological effects, which hold potential for their utilization in the food industry as natural preservatives, antioxidants, or as functional food ingredients [2]. However, polyphenols are prone to degradation under different storage conditions such as temperatures, humidity, light, and pH [3] and also have low bioavailability derived from their low solubility, instability during digestion, and difficult cell membrane diffusion [4]. For this reason, it is of great importance to improve their stability which can be achieved by various microencapsulation techniques.

Spray drying is a widely used method for microencapsulation of bioactive molecules in which the liquid extract with the dissolved carrier is passed through a stream of hot air in which the solvent, evaporates and a powder is formed with bioactive molecules encapsulated in the protective coating of the carrier [5]. The physicochemical properties of powders depend on the applied process parameters, properties of the feed, and the adequate choice of carrier type and the proportion in the mixture [6]. Some of the carriers which are often used in spray drying technology are starch and its derivatives (e.g., maltodextrins and cyclodextrins) and gums (e.g., gum arabic). Maltodextrins (MD) are highly soluble linear polymers obtained by partial hydrolysis of starch consisting of 3–20 β-d-glucose units [7], often mixed with other carriers due to their low emulsifiability [8]. Cyclodextrins (CD), including α-, β- and γ-cyclodextrin, are enzymatically hydrolyzed starch derivatives consisting of a hydrophobic cavity that allows accommodation of various guest molecules and of a hydrophilic external surface providing aqueous solubility [8]. Gum arabic (GA) is a variable complex natural derivative of the acacia plant consisting of a mixture of arabinogalactan, monosaccharides (galactose, rhamnose, arabinose) and glycoprotein [9]. These carriers are widely used since they meet the necessary requirements, including the “generally recognized as safe” (GRAS) status, relatively high water solubility, high molecular weight, and high glass transition temperature, which allow the protection of the final product from external factors such as heat, oxygen, humidity, and light [5]. To our knowledge, there is only one work related to the spray drying of *Laurus nobilis* L. leaf extracts [10] and another on the Mexican laurel leaf (*Litsea glaucescens*) [11], which has similar chemical composition as *Laurus nobilis* L. [12]. The physicochemical characterization of the powders obtained in the mentioned studies did not cover some of the parameters important for the stability and applicability of powders, such as hygroscopicity, moisture content, or solubility. In addition, the individual polyphenolic content or bioaccessibility during different stages of digestion was not investigated, showing the need for further research in order to find optimal process parameters that would result in the highest quality of the powders.

The aim of this research was to examine the influence of inlet temperature (120–180 **°**C), carrier type (β-CD, β-CD + MD 50:50, and β-CD + GA 50:50), and sample:carrier ratio (1:1, 1:2, and 1:3) on the process yield, physicochemical parameters (moisture content, solubility, hygroscopicity) and encapsulation efficiency of spray-dried laurel leaf extract, with the hypothesis that all of the applied process parameters would have significant influence. In addition, microcapsule morphology, antioxidant capacity, polyphenolic profile, and bioaccessibility will be examined in the powders where the highest encapsulation efficiency is obtained.

## 2. Materials and Methods

### 2.1. Chemicals and Reagents

Mili-Q system (Millipore, Bedford, MA, USA) was applied for the purification of distilled water. Kemika d.d. (Zagreb, Croatia) supplied ethanol (96%), methanol, sodium chloride, Fe (III) chloride hexahydrate, sodium acetate (99%), sodium bicarbonate, and HPLC grade formic acid (99%). Sigma Aldrich (St. Louis, MO, USA) supplied maltodextrin (DE 4–7), bile salts, porcine pancreatin, porcine gastric mucosa pepsin, and 2,2-diphenyl-1-(2,4,6- trinitrophenyl) hydrazyl (DPPH), as well as standards of quercetin-3-glucoside, myricetin, gallic, syringic, ferulic, protocatechuic, caffeic, chlorogenic, p-coumaric, and rosmarinic acid. Gum arabic, β-cyclodextrin, Trolox, and 2,4,6-tri(2-pyridyl)-s-triazine (TPTZ) were obtained from Acros Organics (Geel, Belgium). Honeywell Riedel-de-Haën (Charlotte, NC, USA) supplied fluorescein sodium salt, while J.T.Baker (Deventer, The Netherlands) supplied glacial acetic acid, hydrochloric acid (37%), and HPLC grade acetonitrile. Extrasynthese (Genay, France) supplied standards of procyanidin B2, rutin, kaempferol-3-glucoside, luteolin, apigenin, catechin, epigallocatechin gallate, and epicatechin gallate. Methanol (ethanol with 0.5% *v*/*v* DMSO for apigenin) stock solutions of the standards were used to produce five working standard dilutions.

### 2.2. Plant Material

Dry laurel leaves collected in November 2021 in Lovran, Croatia (45°17′48.9408″ N/14°16′20.532″ E) were procured from Šafram d.o.o. (Zagreb, Croatia). Prior to extraction, the leaves were ground in an electric grinder (GT11, Tefal, Rumilly, France) in order to produce a coarse powder. The powdered leaves’ total solids (>95%) were determined by drying at 103 ± 2 °C until a constant mass was achieved [13].

### 2.3. Microwave-Assisted Extraction (MAE)

The MAE of laurel leaf polyphenols was carried out at the sample:solvent ratio 1:6.25 and the previously defined optimal parameters [14]: 50% ethanol as a solvent, temperature 80 °C, microwave power 400 W, and irradiation time 10 min. The extract dry matter (4.74%) was determined by drying to constant mass at 103 ± 2 °C [13], while the total phenolic content of the extract (8300 mg gallic acid equivalents (GAE)/L) was determined spectrophotometrically as previously described [14]. Briefly, 100 μL sample, 200 μL Folin-Ciocalteu reagent, and 2 mL of water were mixed in test tubes, and 1 mL of 20% Na_2_CO_3_ aqueous solution was added after 3 min. The samples were placed in a water bath at 50 °C for 25 min, and the absorbance was read at 765 nm on the UV-1600PC spectrophotometer (VWR, Wayne, PA, USA).

### 2.4. Microencapsulation by Spray Drying

Microencapsulation by spray drying of the laurel leaf extract was carried out as shown in the experimental design (Table 1) on the Büchi Mini Spray Dryer B-290 laboratory device in a closed mode paired with the B295 inert loop (Büchi, Switzerland) working with nitrogen as drying gas. β-CD alone or with the addition of MD or GA (1:1 *w*/*w*) was used as a carrier at varying extract dry matter:carrier ratios (1:1, 1:2, and 1:3, *w*/*w*). An appropriate mass of carrier (4.74 g, 9.48 g, and 14.22 g for 1:1, 1:2, and 1:3 extract dry matter:carrier ratio, respectively) was dissolved in 100 mL of water at 50 **°**C for 30 min on a magnetic stirrer (IKA, Staufen, Germany) and mixed with 100 mL of the laurel leaf extract. During the process of spray drying, aspirator capacity, pump capacity, and nozzle cleaner were kept at 80%, 15%, and level 4, respectively. Three different inlet temperatures were applied: 120 **°**C, 150 **°**C and 180 **°**C with the corresponding outlet temperatures of 70 **°**C, 80 **°**C and 90 **°**C, respectively. The laurel leaf powders were produced in duplicate and hermetically stored at room temperature in plastic containers until further analysis.

### 2.5. Microcapsules’ Characterization

#### 2.5.1. Process Yield

The process yield was calculated using the equation [15]:

(1) $$\mathrm{Process}\;\mathrm{yield}\;(\%)=\frac{m_{p}}{m_{d}+m_{c}}\times 100$$

where *m_p_* is the mass (g) of powder, *m_d_* is the extract dry matter (g) in the volume used for drying, and *m_c_* is the mass of the added carrier (g).

#### 2.5.2. Moisture Content

The powders’ moisture content was analyzed by drying to constant mass at 103 ± 2 **°**C [13].

#### 2.5.3. Solubility

The powders’ solubility was determined using a previously described method [15] and calculated using the following equation:

(2) $$\mathrm{Solubility}\;(\%)=\frac{m_{s}}{m_{p\;}\;}\times 100$$

where *m_s_* is the mass (g) of the dried supernatant, and *m_p_* represents the mass (g) of laurel leaf powder used for analysis.

#### 2.5.4. Hygroscopicity

The hygroscopicity of the obtained powders was determined as previously described [15] and expressed as g of absorbed moisture per 100 g of powder according to the equation:

(3) $$\mathrm{Hygroscopicity}\;(g/100\;g)=\frac{m_{7}-m_{0}}{m_{0}}\times 100$$

where *m*_7_ is the mass (g) of the powder after 7 days, and *m*_0_ is the initial mass (g) of the powder.

#### 2.5.5. Encapsulation Efficiency and Capacity

For the determination of encapsulation efficiency (EE) and capacity (EC), the total and surface polyphenols of the obtained laurel leaf powders were determined spectrophotometrically at 765 nm following a previously described method [14]. The total polyphenols were extracted by mixing 0.2 g of powder with 2 mL of methanol:water:acetic acid (50:42:8) solvent mixture in a test tube stirred briefly on a vortex mixer (IKA, Staufen, Germany). The test tube was then put in an ultrasonic bath for 20 min without heating, centrifuged for 10 min at 3000 rpm, and filtered through Whatman no.40 filter paper. The surface polyphenols were extracted following a similar procedure using methanol:ethanol (50:50) solvent mixture without placing the samples in an ultrasonic bath.

The EE was calculated according to the following equation [16]:

(4) $$\mathrm{EE}=\frac{TPC_{p}}{TPC_{i}}\times 100$$

where *TPC_p_* is the total phenolic content in the obtained powder (mg GAE g^−1^ extract dry matter (DM)), and the *TPC_i_* is the total phenolic content in the initial extract (mg GAE g^−1^ extract DM).

The EC was calculated using the following equation [15]:

(5) $$\mathrm{EC}\;(\%)=\left(\frac{TP-SP}{TP}\right)\times 100$$

where *TP* is the concentration (mg GAE g^−1^ powder) of total polyphenols, and *SP* is the concentration (mg GAE g^−1^ powder) of surface polyphenols.

In order to evaluate the overall encapsulation efficiency of the spray drying process, an overall encapsulation efficiency factor (OEE) was calculated using following equation:

(6) $$\mathrm{OEE}=\frac{EC\times EE}{10,000}$$

where *EC* is the encapsulation capacity (%), and *EE* is the encapsulation efficiency (%). By multiplying the TPC of the initial extract with the OEE, the amount of the successfully encapsulated polyphenols can be predicted.

#### 2.5.6. SEM Analysis

The study of particle size and morphology of the obtained microcapsules was carried out at the Ruđer Bošković Institute, Zagreb, Croatia, on the JSM-7000F high-resolution field emission SEM (scanning electron microscope) (Jeol, Tokyo, Japan). Laurel leaf powders were deposited on carbon tape in a thin layer on a sample holder to fix them in place and enable electrical contact with the instrument. An accelerating voltage of 5000 V at the standard objective-sample distance (10 mm) was applied, and photomicrographs of each sample were taken at 2000× and 5000× magnification using a secondary electron detector.

#### 2.5.7. UPLC-MS^2^ Analysis

For the UPLC-MS^2^ analysis, 1 g of the powders was mixed in a test tube with 10 mL of 80% (*v*/*v*) methanol and placed in an ultrasonic bath for 20 min. The samples were filtered into a 10 mL volumetric flask using the Whatman no.40 filter paper and made up to volume with the solvent. An aliquot of 1.5 mL was filtered into glass vials using 0.45 μm syringe filters and stored at −18 °C until further analysis. The polyphenolic profile of the powders was determined on the Agilent 1290 RRLC UPLC-MS^2^ system paired with 6430 Series LC-MS Triple Quadrupole mass spectrometer (Agilent, Santa Clara, CA, USA) at the conditions described previously, along with identification and quantification procedure [14]. The concentrations of the analyzed polyphenols were expressed as mg/100 g of the powder (mean value ± standard deviation (SD)). All analyses were carried out in duplicate.

#### 2.5.8. Antioxidant Capacity

The antioxidant capacity of the laurel leaf powders dissolved in 80% methanol as described in Section 2.5.7. was determined by DPPH radical scavenging assay, Ferric Reducing Antioxidant Power (FRAP) assay, and Oxygen Radical Absorbance Capacity (ORAC) assay following previously described methodologies [17]. For the DPPH and FRAP, the absorbances were read on a UV-1600PC spectrophotometer (VWR, Wayne, PA, USA) at 517 nm and 593 nm, respectively. For the ORAC (CLARIOstar Microplate Reader, BMG LABTECH, Germany), the fluorescence intensity with the excitation and emission wavelengths of 485 nm and 528 nm, respectively, were monitored during 120 min in intervals of 90 s. The collected data were analyzed using the MARS 2.0 software (BMG LABTECH, Offenburg, Germany). Trolox was used as a standard for all three methods. All measurements were performed in duplicate, and the results were expressed in µmol Trolox equivalents (TE) g^−1^ powder as mean value ± SD.

#### 2.5.9. Bioaccessibility of Polyphenols

The bioaccessibility of polyphenols encapsulated in the powders was examined in a simulated three-step in vitro digestion following a recently described methodology [15]. Briefly, 250 mg of powders (750 μL of laurel leaf extract) was mixed with 800 μL of pepsin solution (40 mg mL^−1^) and 10 mL of 0.9% NaCl solution in 50 mL Falcon tubes. The pH was adjusted to 2 by adding an adequate volume of 0.1M HCl, if necessary. The samples were placed in a water bath shaker (IKA, Staufen, Germany) for 1 h at 100 rpm and a temperature of 37 °C. In order to stop the reaction, the samples were then put on ice for 5 min. Pur-A-Lyzer 6–8 kDa dialysis membranes (Sigma-Aldrich, Steinheim, Germany) containing the mixture of 1 mL of 0.5 M NaHCO_3_ and 1 mL of 0.9% NaCl were added in the Falcon tubes, which were again placed in water bath shaker at the same conditions for 45 min. Afterward, the pH was adjusted to 6.5 by adding 1 M NaHCO_3_ in the necessary volume, 2.5 mL of pancreatin (2 mg mL^−1^)-bile salts (12 mg mL^−1^) solution was added to the reaction, and the samples were incubated for another 2 h in a water bath shaker; 2 mL aliquots of each phase were taken for the UPLC-MS^2^ analysis of the phenolic content performed as described in Section 2.5.7. The process and analysis were performed in duplicate, and the results were expressed in mg g^−1^ extract DM as mean value ± SD.

### 2.6. Statistical Analysis

Statistical analysis of the data was performed using the Statistica ver. 10.0 (Statsoft Inc., Tulsa, OK, USA) software. A full factorial design (Table 1) comprising 27 experimental trials performed in duplicate was applied in order to evaluate the influence of spray drying parameters on the physicochemical properties of the obtained powders. The inlet temperature, type of carrier, and sample:carrier ratio were the independent variables (X) observed at three levels, while moisture content, process yield, solubility, hygroscopicity, encapsulation efficiency, and encapsulation capacity were the dependent variables (Y).

The normality of the data set and homogeneity of the residuals were analyzed by Shapiro–Wilk’s and Levene’s tests, respectively, followed by one-way analysis of variance (ANOVA) paired with Tukey’s HSD multiple comparison test on normally distributed and homogenous data. Nonparametric Kruskal–Wallis one-way ANOVA followed by multiple comparison of mean ranks were applied to the data that were not normally distributed and/or homogenous. All of the tests were considered significant at *p* ≤ 0.05.

## 3. Results and Discussion

This study evaluated the influence of drying temperature, applied carrier, and sample:carrier ratio on different properties of the obtained powders relevant to their stability during storage and biological activity. The experimental design and obtained values of observed parameters are shown in Table 1, while the results of the statistical analysis are shown in Table 2.

### 3.1. Process Yield

When the process yield is higher than 50%, the process of spray drying can be considered successful [18]. As shown in Table 1, the process yield in this study ranged from 66.20 to 84.46% showing that the process was successful at all of the applied drying conditions. Statistical analysis (Table 2) showed that none of the applied conditions had a significant influence on the process yield. This is in agreement with made observations that all produced powders were in a free-flowing form, and stickiness or adherence to chamber walls did not occur at any of the drying conditions applied. Obtained variations in yield may be a result of manual collecting of the particles adhered to the cyclone wall, as well as loss of the fine particles through the outlet air filter [19].

### 3.2. Moisture Content

Moisture content is a highly relevant requirement for the stability of the obtained powders during packaging and storage. A moisture content lower than 5% is desirable since there is a lower chance of microbial growth, the solubility of powders is higher, and overall stability is greater, making the powders applicable in the industry [5]. As shown in Table 1, the moisture content in this study ranged from 2.26 to 4.72% showing that all of the obtained powders have the moisture content required for their stability. However, statistical analysis (Table 2) showed that the powders obtained at inlet temperatures above 150 °C had a significantly (*p* < 0.01) lower moisture content. This is expected since the temperature gradient between the drying air and the atomized particles is greater at higher inlet temperatures resulting in increased water evaporation [5]. The type of carrier and the sample:carrier ratio had no statistically significant influence on the moisture content.

### 3.3. Solubility

Solubility is an important quality factor influencing the reconstitution behavior of the powders, and low solubility can cause difficulties during production of the enriched products [5]. The solubility of the powders obtained in the present study ranged widely from 28.20 to 60.15% (Table 1). The statistical analysis showed that only the type of the applied carrier had a statistically significant influence (*p* < 0.01) on the solubility and that the powders obtained using a combination of β-CD with either MD or GA resulted in higher solubility than when β-CD was used alone. This can be explained by the low water solubility of β-CD, which was enhanced by the presence of more water-soluble MD and GA [8].

### 3.4. Hygroscopicity

Hygroscopicity is a parameter that shows how much moisture the powder absorbs from a relatively humid environment over a certain time period and, as such, can be a valuable predictor of the powder’s stability during storage. In the present study, the hygroscopicity of the powders during 7 days ranged from 9.94 to 21.8% (Table 1). Type of carrier and sample:carrier ratio significantly (*p* < 0.01) influenced the hygroscopicity, while the influence of temperature was insignificant (Table 2). Powders obtained using β-CD alone or in combination with MD had a significantly lower hygroscopicity than the powders obtained using the combination of β-CD + GA 50:50. This can be explained by the branched structure of GA, which allows water molecules to bind to the hydroxyl groups in the chains [20], while MD (DE 4–7) is less polymerized and the β-CD has a specific cyclic structure with hydrophobic cavity and a hydrophilic outer part and are therefore less susceptible for the binding of water molecules [8]. The sample:carrier ratio of 1:1 resulted in a higher hygroscopicity than both 1:2 and 1:3 ratios which can be explained by the increased dry matter content with the addition of carrier and consequently lower water content and lower hygroscopicity [21].

### 3.5. Encapsulation Efficiency and Capacity

Encapsulation efficiency of the spray drying process wascalculated from the TPC of the initial extract (Supplementary Materials; Table S1), and the TPC of the obtained powders ranged from 45.3 to 92.07% with the mean value of 73.54% (Table 1), which is comparable to the range of 72.9–99.3% [10], as well as the value of 70% [11] achieved during spray drying of laurel leaf (*L. nobilis* L.) and Mexican laurel leaf (*Litsea glaucescens*) extract, respectively. Statistical analysis (Table 2) showed that inlet temperature had no statistically significant influence on the encapsulation efficiency, while the applied carrier and sample:carrier ratio had a significant (*p* < 0.01) influence. The highest encapsulation efficiency was achieved when β-CD was applied as a carrier, possibly due to the structure of β-CD whose hydrophobic central cavity diameter was shown to be suitable for stable binding with flavonoids [22] whose β-ring showed high affinity for binding with β-CD [23]. Encapsulation capacity calculated using the concentration of total and surface polyphenols (Supplementary Materials; Table S1) ranged from 34.26 to 80.23%. Statistical analysis showed that inlet temperature and the sample:carrier ratio had a significant influence (*p* < 0.01) on the encapsulation capacity, while the applied carrier was not a significant parameter. The highest encapsulation capacity was obtained at the temperature of 180 °C, possibly due to faster drying rates which allowed the early structural formation of the complexes and therefore resulted in more efficient entrapment of the polyphenols [24]. The sample:carrier ratio of 1:2 resulted in both the highest encapsulation efficiency and capacity. The ratio of 1:2 was likely more efficient than the 1:1 ratio due to the higher concentration of the carrier, which was allowed to precipitate faster on the surface of the dispersed phase and therefore prevented the diffusion of polyphenols across the phase boundary [25]. Further addition of the carrier at the ratio of 1:3 possibly shortened the time of contact of the feed with drying air which slowed heat and mass transfer and delayed structural formation of the complexes resulting in less efficient entrapment.

The overall efficiency factor calculated from the encapsulation efficiency and capacity values showed that the highest overall encapsulation efficiency of the spray drying process could be achieved at the inlet temperature of 180 °C by using any of the three applied carriers at the ratio of 1:2. The use of β-CD + MD 50:50, however, would result in the most desirable physicochemical properties of the powder since it had a higher solubility than when only β-CD was applied and a lower hygroscopicity compared to β-CD + GA 50:50. Nevertheless, in order to observe the influence of carrier on other properties of the powders, the powders obtained using all three carriers at the temperature of 180 °C and the sample:carrier ratio 1:2 (powder samples 22, 23 and 24) were chosen for further analysis of the particle size by SEM, individual polyphenolic content by UPLC-MS^2^, antioxidant capacity, and the in vitro bioavailability.

### 3.6. Morphology of the Microcapsules

**Figure 1:**
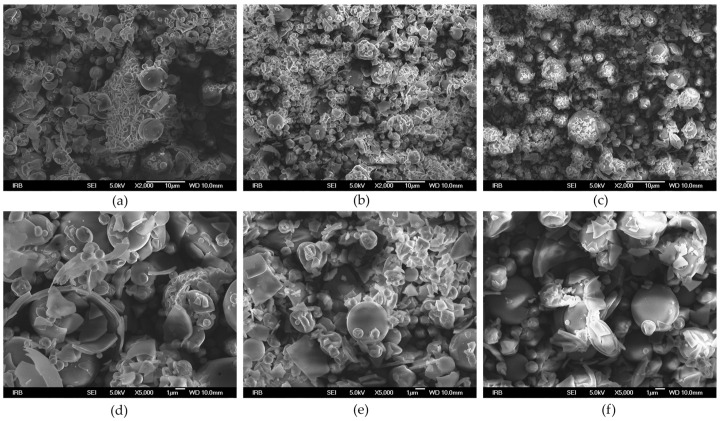
SEM images of the laurel leaf powders obtained at 180 °C and 1:2 sample:carrier ratio using different carriers: (**a**,**d**) β-CD; (**b**,**e**) β-CD + MD 50:50; (**c**,**f**) β-CD + GA 50:50.

In order to observe the morphology of the microcapsules as influenced by the type of carrier, the powders obtained at 180 °C and a carrier ratio 1:2 were analyzed by SEM at 2000× (Figure 1a–c) and 5000× (Figure 1d–f) magnification. None of the carriers resulted in uniform microcapsules whose size ranged from 1 to 8μm in all powders.

Following a classification established by Walton (2000) [26], the type of microcapsules obtained by all the carriers can be classified as skin-forming with a visible mixture of non-broken microcapsules, fractured spheres, and broken shells characteristic for drying at high inlet temperatures that lead to rapid evaporation of the solvent [27].

### 3.7. Individual Polyphenolic Content

UPLC-MS^2^ analysis was performed on the laurel leaf powders obtained at the temperature of 180 °C and sample:carrier ratio 1:2 in order to investigate the influence of the carrier type on the powders’ polyphenolic profile (Table 3). The compounds were identified and quantified as described previously [14], and the chromatograms are shown in Supplementary Materials (Figure S1). In total, twenty-nine polyphenols were identified in all powders, among which ten were phenolic acids represented largely by syringic acid, four flavones (mainly luteolin), four flavan-3-ols (mostly catechin and epicatechin in equal amounts), one proanthocyanidin (B-type procyanidin trimer) and ten flavonols (dominantly quercetin glycosides) which were the most abundant group representing around 74% of all polyphenols in each powder which is in accordance with the polyphenolic content of laurel leaf extracts [1].

Statistical analysis showed that carrier type significantly (*p* < 0.05) influenced the individual polyphenolic content of the powders. All polyphenolic groups and total polyphenols were more abundant in powders obtained using β-CD or the combination of β-CD + MD 50:50 compared to powders obtained using β-CD + GA 50:50. The largest differences were observed in the concentration of flavonols, namely quercetin-3-glucoside, and isorhamnetin-3-hexoside. Even though the branched structure of GA may facilitate the binding of polyphenols due to more binding sites available for the interaction, the presence of neutral sugars in the structure of GA [28] may result in the steric hindrance of the adsorption as it was previously shown in the case of pectins and procyanidins [29]. In addition, the degree of glycosylation and type of hydroxylation of the glycosides might have affected the solubility of flavonol glycosides and therefore hindered the binding with the complex structure [30] of GA, resulting in lower content than with the other two carriers.

### 3.8. Antioxidant Capacity

In order to examine the influence of carrier on the antioxidant capacity of the powders, powders obtained at 180 °C and sample:carrier ratio 1:2 were analyzed by DPPH, FRAP, and ORAC assay, and the results are shown in Table 4.

As can be observed, all three assays showed that the powder obtained using β-CD + MD 50:50 showed the highest antioxidant capacity. These results indicate that not only the content of polyphenols was responsible for the antioxidant capacity since the powders obtained by using β-CD and β-CD + MD 50:50 had the same concentration of polyphenols as determined by UPLC-MS^2^. It is likely that other antioxidant compounds, such as fatty acids or chlorophyll present in the laurel leaves [1], were encapsulated more efficiently by the combination of β-CD and MD due to their different chemical structures, which allow diverse binding mechanisms. In support, it was shown that combinations of carriers often result in higher antioxidant capacity of the encapsulated plant extracts, and maltodextrin was shown to be more efficient than GA in the encapsulation of chlorophyll [31].

### 3.9. Bioaccessibility of Polyphenols

**Figure 2:**
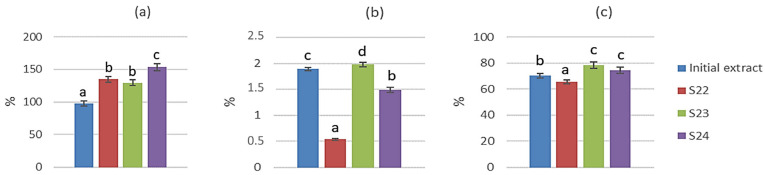
Bioaccessibility of laurel leaf flavonols in the initial extract and powders obtained using β-CD (S22), β-CD + MD 50:50 (S23), and β-CD + GA 50:50 (S24) during (**a**) gastric phase of in vitro digestion; (**b**) absorbed phase of in vitro digestion; (**c**) intestinal phase of in vitro digestion. Columns marked with different letters within picture are statistically different at *p* < 0.05.

The bioavailability of polyphenols is defined as the rate and degree of their absorption through the epithelial cells in the gastrointestinal tract, and it includes bioaccessibility (release) of compounds from the food matrix and bioactivity (digestion, absorption, metabolism, distribution, and the physiological response). Since the determination of bioavailability is complex due to ethical issues and impracticality, most of the research is focused on the bioaccessibility of bioactive molecules as the first step and key factor in predicting the bioavailable fraction of the compounds [32]. In order to observe the influence of carriers applied during spray drying on the bioavailability of laurel leaf polyphenols, the initial extract and the powders obtained at the temperature of 180 °C and sample:carrier ratio 1:2 with all three carriers (β-CD (S22); β-CD + MD 50:50 (S23); β-CD + GA 50:50 (S24) were analyzed by UPLC-MS^2^. The content of flavonols as the most abundant polyphenolic group was monitored through three stages of in vitro digestion and expressed as a percentage of the concentration in the initial extract/powders (Figure 2a–c). The individual content of flavonols is shown in Supplementary Materials (Table S2).

As it can be observed, during the gastric phase of in vitro digestion (Figure 2a), the concentration of the flavonols ranged between ~100 and 150% compared to the content in the initial extract and powders. The bioaccessible portion from the initial extract was the lowest, but the polyphenols did not degrade, which is consistent with the data on their stability in the acidic medium [33]. On the other hand, the percentages higher than 100% for the bioaccessible flavonols in powders might be a result of the breaking of the bonds between the carriers and flavonols due to the acidic medium, which enhances the release of polyphenols from the food matrix in general [34]. There was no significant difference between the polyphenolic content in powders obtained using β-CD or β-CD + MD 50:50, while the highest percentage was released from the powder using β-CD + GA 50:50. The absorbed portion of flavonols (Figure 2b) from the intestinal phase ranged between ~0.5 and 2% which is consistent with the literature data which states that less than 10% of polyphenols are absorbed during the intestinal phase of digestion [32], while the majority is a substrate to colon microbiota which produces various metabolites which, when absorbed, potentially possess higher biological potential than their parent compounds [35]. The β-CD + MD 50:50 carrier resulted in the highest absorbed percentage, whereas the use of β-CD resulted in the lowest absorbed percentage of flavonols. In the intestinal phase, the percentage of bioaccessible flavonols (Figure 2c) was significantly lower than in the gastric phase, ranging from ~62 to 80%, which is consistent with previous findings where the largest portion of polyphenols was released during the gastric phase of digestion, and a significant degradation occurred in the intestinal phase [36]. This might be a result of a change of pH from acidic to mildly alkaline in the duodenum, as alkaline pH often causes oxidation and degradation of polyphenols [33]. Encapsulation using β-CD + MD 50:50 or β-CD + GA 50:50 preserved a higher percentage of flavonols compared to the initial extract, while the β-CD alone preserved a lower percentage than the initial extract. These results indicate that applying a combination of carriers is the most efficient way to achieve the stability of laurel leaf flavonols during digestion which is likely due to the difference in their structure allowing more interaction and binding sites for the polyphenols [32]. Overall, spray drying increased the bioaccessibility of laurel leaf flavonols during three different stages of in vitro digestion and showed potential for increasing their bioavailability. Further research, including the colon stage of digestion, as well as investigation of the metabolites’ fate in plasma, would provide a detailed insight into the bioavailability of laurel leaf flavonols and their fate in the human body.

## 4. Conclusions

This study emphasized the importance of optimization for the microencapsulation of laurel leaf polyphenols by spray drying since the physicochemical characteristics depended on the applied process parameters. It was shown that the highest encapsulation efficiency could be obtained by using either of the three applied carrier mixtures at a ratio of 1:2 and the temperature of 180 °C, while the most desirable solubility and hygroscopicity were achieved using β-CD + MD 50:50. None of the parameters influenced the process yield, while the moisture content depended only on the inlet temperature and was optimal at temperatures above 150 °C. A total of 29 polyphenols were identified in the powders, with flavonols being the dominant group. The individual polyphenolic content was higher when β-CD alone or in combination with MD was applied as a carrier, while β-CD + MD 50:50 carrier combination provided the highest antioxidant capacity. The in vitro digestion showed that microencapsulation by spray drying increased the bioaccessibility of laurel flavonols, demonstrating the potential to enhance their bioavailability in vivo. The use of a carrier mixture (β-CD + MD/GA 50:50) was more efficient in preserving the laurel leaf flavonols during digestion than using only β-CD. Based on the findings of this study, it can be concluded that microencapsulation by spray drying is a promising technique for the stabilization of the laurel leaf polyphenols during storage and digestion, thus enabling efficient utilization of their potential as functional food ingredients.

## Figures and Tables

**Table 1:** Physicochemical properties, encapsulation efficiency, and capacity of laurel leaf powders obtained at different inlet temperatures using different carriers at varying ratios.

Sample	Inlet Temperature	Carrier	Sample:Carrier Ratio	Moisture Content %	Process Yield %	Solubility %	Hygroscopicity mg/100 g	Encapsulation Efficiency %	Encapsulation Capacity %	OEE
1	120	β-CD	1:1	2.69 ± 0.13	77.77 ± 0.55	28.02 ± 0.20	17.23 ± 0.12	84.71 ± 0.28	42.55 ± 2.06	0.36 ± 0.02
2		β-CD + MD 50:50		3.54 ± 0.08	75.69 ± 0.53	57.83 ± 0.41	17.82 ± 0.13	69.07 ± 1.13	42.20 ± 0.63	0.29 ± 0.00
3		β-CD + GA 50:50		3.53 ± 0.35	75.50 ± 0.53	53.86 ± 0.38	21.80 ± 0.15	71.26 ± 0.92	53.22 ± 0.00	0.38 ± 0.00
4		β-CD	1:2	4.58 ± 0.14	78.91 ± 0.56	44.85 ± 0.32	12.70 ± 0.09	82.20 ± 1.19	55.46 ± 0.99	0.46± 0.01
5		β-CD + MD 50:50		4.54 ± 0.24	75.99 ± 0.53	56.20 ± 0.40	15.16 ± 0.11	78.50 ± 1.72	69.46 ± 0.67	0.55 ± 0.01
6		β-CD + GA 50:50		4.72 ± 0.08	71.69 ± 0.50	53.01 ± 0.37	18.30 ± 0.13	81.98 ± 2.61	76.67 ± 1.70	0.63 ± 0.03
7		β-CD	1:3	5.71 ± 0.02	73.70 ± 0.52	45.67 ± 0.32	9.94 ± 0.07	72.40 ± 1.59	38.55 ± 3.49	0.28 ± 0.03
8		β-CD + MD 50:50		5.44 ± 0.21	70.36 ± 0.50	46.04 ± 0.32	11.83 ± 0.08	53.09 ± 0.86	56.79 ± 1.07	0.30 ± 0.01
9		β-CD + GA 50:50		4.39 ± 0.35	84.46 ± 0.59	47.33 ± 0.33	17.59 ± 0.12	50.20 ± 0.71	62.70 ± 1.39	0.31 ± 0.00
10	150	β-CD	1:1	3.70 ± 0.22	72.09 ± 0.51	40.47 ± 0.28	18.02 ± 0.13	63.48 ± 0.29	34.26 ± 0.19	0.22± 0.00
11		β-CD + MD 50:50		3.95 ± 0.15	77.28 ± 0.54	58.90 ± 0.41	16.35 ± 0.12	61.91 ± 0.79	35.91 ± 2.09	0.22 ± 0.01
12		β-CD + GA 50:50		2.70 ± 0.16	73.71 ± 0.52	54.77 ± 0.39	19.46 ± 0.14	60.34 ± 0.84	47.22 ± 3.02	0.29 ± 0.02
13		β-CD	1:2	3.66 ± 0.16	73.73 ± 0.52	45.00 ± 0.32	12.08 ± 0.08	88.67 ± 1.60	62.33 ± 1.47	0.55 ± 0.00
14		β-CD + MD 50:50		2.80 ± 0.17	69.87 ± 0.49	46.03 ± 0.32	15.31 ± 0.11	69.87 ± 5.61	61.65 ± 1.50	0.43 ± 0.05
15		β-CD + GA 50:50		3.84 ± 0.10	67.91 ± 0.48	33.03 ± 0.23	19.80 ± 0.14	83.01 ± 3.39	67.29 ± 0.57	0.56 ± 0.02
16		β-CD	1:3	3.37 ± 0.16	66.20 ± 0.47	47.35 ± 0.33	10.70 ± 0.08	81.84 ± 0.42	41.99 ± 3.18	0.34 ± 0.02
17		β-CD + MD 50:50		2.26 ± 0.17	81.58 ± 0.57	52.36 ± 0.37	13.55 ± 0.10	78.76 ± 1.12	69.67 ± 2.60	0.55 ± 0.03
18		β-CD + GA 50:50		2.72 ± 0.18	80.28 ± 0.56	52.66 ± 0.37	18.08 ± 0.13	74.95 ± 2.93	59.67 ± 3.22	0.45 ± 0.04
19	180	β-CD	1:1	3.65 ± 0.12	68.96 ± 0.49	39.81 ± 0.28	20.15 ± 0.14	81.44 ± 1.72	62.46 ± 1.92	0.51 ± 0.03
20		β-CD + MD 50:50		3.73 ± 0.24	75.14 ± 0.53	58.91 ± 0.41	18.46 ± 0.13	71.18 ± 2.04	59.82 ± 0.92	0.43 ± 0.02
21		β-CD + GA 50:50		3.83 ± 0.22	73.10 ± 0.51	52.63 ± 0.37	21.27 ± 0.15	67.26 ± 0.29	55.88 ± 1.59	0.38± 0.01
22		β-CD	1:2	3.42 ± 0.16	74.69 ± 0.53	47.45 ± 0.33	14.28 ± 0.10	89.83 ± 1.03	67.96 ± 1.46	0.61 ± 0.02
23		β-CD + MD 50:50		2.51 ± 0.26	78.96 ± 0.56	59.47 ± 0.42	14.13 ± 0.10	74.41 ± 3.39	80.23 ± 1.70	0.60 ± 0.04
24		β-CD + GA 50:50		3.49 ± 0.27	75.67 ± 0.53	52.88 ± 0.37	17.99 ± 0.13	75.38 ± 0.62	69.44 ± 0.94	0.52 ± 0.01
25		β-CD	1:3	3.50 ± 0.04	74.38 ± 0.52	45.88 ± 0.32	12.00 ± 0.08	92.07 ± 0.57	58.58 ± 0.86	0.54 ± 0.00
26		β-CD + MD 50:50		3.66 ± 0.17	76.39 ± 0.54	60.15 ± 0.42	15.24 ± 0.11	45.30 ± 2.15	59.17 ± 1.31	0.27 ± 0.01
27		β-CD + GA 50:50		4.39 ± 0.19	73.35 ± 0.52	46.12 ± 0.32	16.41 ± 0.12	82.63 ± 0.85	75.89 ± 1.71	0.63 ± 0.02
Average				3.72	74.72	49.14	16.14	73.54	58.04	0.43

β-CD = β-cyclodextrin; MD = maltodextrin; GA = gum arabic. OEE = overall encapsulation efficiency factor. Results are expressed as mean ± SD.

**Table 2:** Influence of spray drying parameters on the physicochemical characteristics of the obtained laurel leaf powders.

Source of Variation	N	Process Yield %	Moisture Content %	Solubility %	Hygroscopicity g/100 g	EE %	EC %	OEE
Inlet temperature		*p* = 0.27 ‡	*p* < 0.01 †	*p* = 0.31 ‡	*p* = 0.70 ‡	*p* = 0.59 ‡	*p* < 0.05 †	*p* < 0.05 †
120 °C	18	76.01 ± 0.95 ^a^	4.35 ± 0.22 ^b^	48.09 ± 2.04 ^a^	15.82 ± 0.77 ^a^	71.49 ± 2.86 ^a^	55.29 ± 2.97 ^a^	0.40 ± 0.03 ^a^
150 °C	18	73.63 ± 1.22 ^a^	3.22 ± 0.14 ^a^	47.84 ± 1.81 ^a^	15.93 ± 0.77 ^a^	73.65 ± 2.38 ^a^	53.33 ± 3.14 ^a^	0.40 ± 0.03 ^a^
180 °C	18	74.52 ± 0.63 ^a^	3.58 ± 0.12 ^a^	51.48 ± 1.64 ^a^	16.66 ± 0.77 ^a^	75.50 ± 3.21 ^a^	65.49 ± 1.94 ^b^	0.50 ± 0.03 ^b^
Carrier		*p* = 0.22 ‡	*p* = 0.76	*p* < 0.01 †	*p* < 0.01 †	*p* < 0.01†	*p* < 0.05 †	*p* = 0.38 ‡
β-CD	18	73.38 ± 0.96 ^a^	3.81 ± 0.02 ^a^	42.72 ± 1.41 ^a^	14.12 ± 0.81 ^a^	81.84 ± 2.07 ^b^	51.57 ± 2.83 ^a^	0.43 ± 0.03 ^a^
β-CD + MD 50:50	18	75.70 ± 0.96 ^a^	3.60 ± 0.02 ^a^	55.10 ± 1.29 ^b^	15.32 ± 0.47 ^a^	66.90 ± 2.65 ^a^	59.43 ± 3.14 ^a^	0.40 ± 0.03 ^a^
β-CD + GA 50:50	18	75.08 ± 0.96 ^a^	3.73 ± 0.02 ^a^	49.59 ± 1.57 ^b^	18.97 ± 0.40 ^b^	71.89 ± 2.56 ^a^	63.10 ± 2.33 ^b^	0.46 ± 0.03 ^a^
Sample:Carrier Ratio		*p* = 0.79 ‡	*p* = 0.61 ‡	*p* = 0.67 ‡	*p* < 0.01 †	*p* < 0.01 †	*p* < 0.01 †	*p* < 0.01 †
1:1	18	74.36 ± 0.63 ^a^	3.50 ± 0.11 ^a^	49.47 ± 2.48 ^a^	18.95 ± 0.42 ^b^	70.07 ± 1.92 ^a^	48.17 ± 2.35 ^a^	0.34 ± 0.02 ^a^
1:2	18	74.16 ± 0.87 ^a^	3.73 ± 0.18 ^a^	48.66 ± 1.79 ^a^	15.53 ± 0.60 ^a^	80.43 ± 1.57 ^b^	67.83 ± 1.75 ^b^	0.54 ± 0.02 ^b^
1:3	18	75.63 ± 1.31 ^a^	3.94 ± 0.27 ^a^	49.28 ± 1.12 ^a^	13.93 ± 0.69 ^a^	70.14 ± 3.78 ^a^	58.11 ± 2.73 ^a^	0.41 ± 0.03 ^a^
Total	54							

N = number of trials. β-CD = β-cyclodextrin. MD = maltodextrin. GA = gum arabic. EE = encapsulation efficiency; EC = encapsulation capacity; OEE = overall encapsulation efficiency factor Results are expressed as mean ± SE. Values with different letters within a parameter are statistically different at *p* ≤ 0.05. † The variable is statistically significant at *p* ≤ 0.05. ‡ The variable is statistically insignificant at *p* ≤ 0.05.

**Table 3:** Individual polyphenolic content of laurel leaf powders obtained with different carriers at 180 °C and sample:carrier ratio 1:2 as determined by UPLC-MS^2^.

Compound Number	Retention Time	Tentative Identification	Concentration (mg 100 g^−1^ Powder)		
			β-CD	β-CD + MD 50:50	β-CD + GA 50:50
	Phenolic acids				
**1**	1.679	Gallic acid *	0.26 ± 0.01 ^b^	0.18 ± 0.01 ^a^	0.34 ± 0.01 ^c^
**2**	2.313	3,4-dihydrobenzoic acid hexoside	0.17 ± 0.00 ^b^	0.17 ± 0.00 ^b^	0.13 ± 0.00 ^a^
**3**	3.488	Syringic acid *	5.42 ± 0.15 ^b^	5.39 ± 0.15 ^b^	4.35 ± 0.12 ^a^
**4**	3.508	Protocatechuic acid *	0.69 ± 0.02 ^b^	0.60 ± 0.02 ^a^	0.54 ± 0.02 ^a^
**5**	4.259	Rosmarinic acid *	0.55 ± 0.02 ^b^	0.78 ± 0.02 ^c^	0.44 ± 0.01 ^a^
**6**	4.813	*p*-hydroxybenzoic acid	0.45 ± 0.01 ^a^	0.49 ± 0.01 ^a^	0.44 ± 0.01 ^a^
**7**	5.043	Chlorogenic acid *	0.38 ± 0.01 ^b^	0.35 ± 0.01 ^b^	0.27 ± 0.01 ^a^
**8**	5.711	Caffeic acid *	0.52 ± 0.01 ^b^	0.19 ± 0.01 ^a^	0.80 ± 0.02 ^c^
**9**	7.28	*p*-coumaric acid *	0.62 ± 0.02 ^b^	0.55 ± 0.02 ^a^	0.50 ± 0.01 ^a^
**10**	8.587	Ferulic acid *	0.79 ± 0.02 ^b^	0.66 ± 0.02 ^a^	0.61 ± 0.02 ^a^
		∑ Phenolic acids	9.84 ± 0.28 ^b^	9.36 ± 0.26 ^b^	8.44 ± 0.24 ^a^
	Flavones				
**11**	2.755	Apigenin-6-C-(*O*-deoxyhexosyl)-hexoside	0.00 ± 0.00 ^a^	0.01 ± 0.00 ^a^	0.01 ± 0.00 ^a^
**12**	6.938	Luteolin-6-C-glucoside	0.54 ± 0.02 ^b^	0.49 ± 0.01 ^b^	0.38 ± 0.01 ^a^
**13**	8.29	Apigenin *	0.07 ± 0.00 ^b^	0.06 ± 0.00 ^a^	0.05 ± 0.00 ^a^
**14**	9.849	Luteolin *	23.22 ± 0.66 ^b^	22.94 ± 0.65 ^b^	16.35 ± 0.46 ^a^
		∑ Flavones	23.84 ± 0.67 ^b^	23.50 ± 0.66 ^b^	16.79 ± 0.47 ^a^
	Flavan-3-ols				
**15**	5.93	Catechin *	124.96 ± 3.53 ^b^	118.19 ± 3.34 ^b^	103.76 ± 2.93 ^a^
**16**	5.937	Epicatechin	123.25 ± 3.49 ^b^	116.71 ± 3.30 ^b^	102.97 ± 2.91 ^a^
**17**	6.02	Epigallocatechin gallate *	0.04 ± 0.00 ^a^	0.08 ± 0.00 ^b^	0.04 ± 0.00 ^a^
**18**	7.905	Epicatechin gallate *	0.22 ± 0.01 ^c^	0.10 ± 0.00 ^b^	0.07 ± 0.00 ^a^
		∑ Flavan-3-ols	248.48 ± 7.03 ^b^	235.09 ± 6.65 ^b^	206.85 ± 5.85 ^a^
	Proanthocyanidins				
**19**	6.249	Procyandinin trimer	78.67 ± 2.23 ^a,b^	80.41 ± 2.27 ^b^	71.30 ± 2.02 ^a^
		∑ Proanthocyanidins	78.67 ± 2.23 ^b^	80.41 ± 2.27 ^b^	71.30 ± 2.02 ^a^
	Flavonols				
**20**	7.692	Rutin *	136.91 ± 3.87 ^a^	125.99 ± 3.56 ^a^	123.21 ± 3.48 ^a^
**21**	7.969	Quercetin-3-glucoside	362.32 ± 10.25 ^b^	358.52 ± 10.14 ^b^	268.95 ± 7.61 ^a^
**22**	8.48	Kaempferol-3-rutinoside	46.60 ± 1.32 ^b^	47.44 ± 1.34 ^b^	33.24 ± 0.94 ^a^
**23**	8.51	Kaempferol-3-hexoside	85.64 ± 2.42 ^a^	82.03 ± 2.32 ^a^	85.67 ± 2.42 ^a^
**24**	8.52	Quercetin-3-pentoside	84.33 ± 2.39 ^a^	82.73 ± 2.34 ^a^	81.42 ± 2.30 ^a^
**25**	8.877	Isorhamnetin-3-hexoside	125.78 ± 3.56 ^b^	122.43 ± 3.46 ^b^	86.69 ± 2.45 ^a^
**26**	8.897	Quercetin-3-rhamnoside	162.36 ± 4.59 ^b^	160.58 ± 4.54 ^b^	133.26 ± 3.77 ^a^
**27**	9.178	Kaempferol-3-*O*-pentoside	38.03 ± 1.08 ^a^	35.19 ± 1.00 ^a^	35.07 ± 0.99 ^a^
**28**	9.825	Kaempferol-3-*O*-deoxyhexoside	0.09 ± 0.00 ^a^	0.09 ± 0.00 ^a^	0.08 ± 0.00 ^a^
**29**	12.137	Myricetin *	0.19 ± 0.01 ^a^	0.19 ± 0.01 ^a^	0.22 ± 0.01 ^b^
		∑ Flavonols	1042.25 ± 29.48 ^b^	1015.19 ± 28.71 ^b^	847.81 ± 23.98 ^a^
		Total	1403.07 ± 39.68 ^b^	1363.54 ± 38.57 ^b^	1151.19 ± 32.56 ^a^

β-CD = β-cyclodextrin; MD = maltodextrin; GA = gum arabic. Results are expressed as mean ± SD. Values within rows marked with different letters were statistically different at *p* < 0.05. * identification confirmed using authentic standards.

**Table 4:** Antioxidant capacity of the laurel leaf powders obtained at 180 °C and a sample:carrier ratio 1:2.

Carrier	DPPH μmol TE g^−1^ Powder	FRAP μmol TE g^−1^ Powder	ORAC μmol TE g^−1^ Powder
β-CD	162.18 ± 4.83 ^a^	210.00 ± 9.06 ^a^	88.59 ± 1.84 ^a^
β-CD + MD 50:50	201.43 ± 3.85 ^b^	267.18 ± 1.93 ^b^	157.92 ± 3.28 ^c^
β-CD + GA 50:50	159.30 ± 1.80 ^a^	196.15 ± 16.77 ^a^	99.43± 2.06 ^b^

The results are expressed as mean ± SD. Values marked with different letters within a column are statistically different at *p* ≤ 0.05.

## Data Availability

The data presented in this study are available on request from the corresponding author.

## Supplementary Materials

The following supporting information can be downloaded at: https://www.mdpi.com/article/10.3390/foods12091923/s1, Figure S1: UPLC-MS/MS chromatogram in MRM acquisition mode of encapsulated laurel leaf extracts obtained at 180 °C and 1:2 sample:carrier ratio and (**a**) β-CD, (**b**) β-CD + MD (50:50) and (**c**) β-CD + GA (50:50).; Table S1: Concentrations of total and surface polyphenols in laurel leaf powders; Table S2: Concentration of laurel leaf flavonols in selected powders and the initial extract during in vitro digestion.
