# Supplementary material for: Physicochemical Properties, Antioxidant Capacity, and Bioavailability of Laurus nobilis L. Leaf Polyphenolic Extracts Microencapsulated by Spray Drying
Source: Foods. 2023 May 8;12(9):1923. doi: 10.3390/foods12091923 (PMC10177897; doi:10.3390/foods12091923)
Supplement: Supplementary file 1 [file foods-12-01923-s001.zip › Figure S1.pdf]

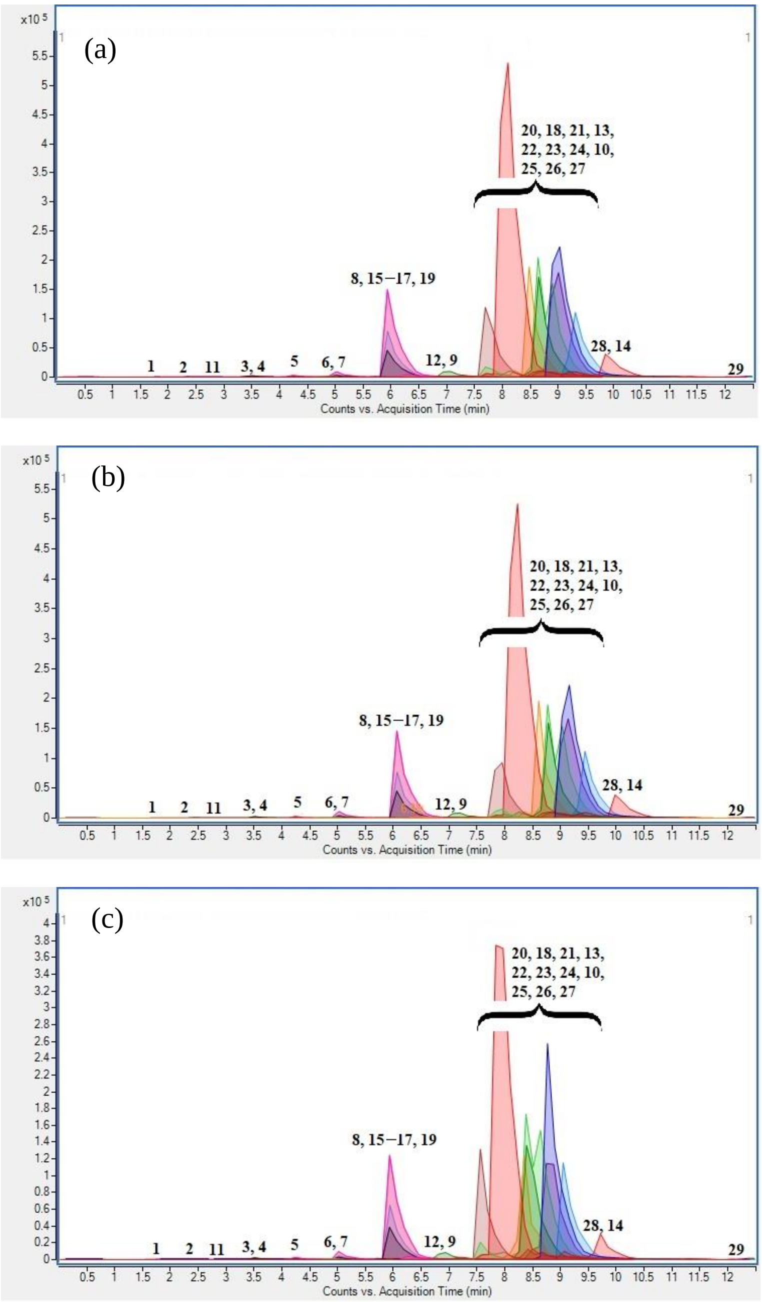

Figure S1 UPLC-MS/MS chromatogram in MRM acquisition mode of encapsulated laurel leaf extracts obtained at 180°C and 1:2 sample:carrier ratio and (**a**) β-CD, (**b**) β-CD + MD (50:50) and (**c**) β-CD + GA (50:50). (1) gallic acid, (2) 3,4-dihidrobenzoic acid hexoside, (3) syringic acid, (4) protocatechuic acid, (5) rosmarinic acid, (6) *p*-hydroxybenzoic acid, (7) chlorogenic acid, (8) caffeic acid, (9) p-coumaric acid, (10) ferulic acid, (11) apigenin-6-C-(Odeoxyhexosyl)-hexoside, (12) luteolin-6-C-glucoside, (13) apigenin, (14) luteolin, (15) catechin. (16) epicatechin, (17) epigallocatechin gallate, (18) epicatechin gallate, (19) procyanidin trimer, (20) rutin, (21) quercetin-3-glucoside, (22) kaempferol-3-rutinoside, (23) kaempferol-3-O-hexoside, (24) quercetin-3-pentoside, (25) isorhamnetin-3-hexoside, (26) quercetin-3-rhamnoside, (27) kaempferol-3-O-pentoside, (28) kaempferol-3-O-deoxyhexoside, (29) myricetin.
